# Association between Hp infection and serum uric acid to high-density lipoprotein cholesterol ratio in adults

**DOI:** 10.3389/fmed.2025.1509269

**Published:** 2025-02-13

**Authors:** Zihan Qin, Yinuo Fang, Yifei Liu, Lingye Zhang, Ruoyi Zhang, Shutian Zhang

**Affiliations:** ^1^Department of Rheumatology and Immunology, Chifeng Cancer Hospital, Chifeng, Inner Mongolia Autonomous Region, China; ^2^College of Basic Medical Sciences, Hebei Medical University, Shijiazhuang, China; ^3^Department of Gastroenterology, Beijing Friendship Hospital, Capital Medical University, Beijing, China; ^4^State Key Laboratory of Digestive Health, National Clinical Research Center for Digestive Diseases, Beijing, China

**Keywords:** Hp, Hp infection, serum uric acid to high-density lipoprotein cholesterol ratio, serum uric acid, high-density lipoprotein cholesterol, adults

## Abstract

**Background:**

*Helicobacter pylori* (Hp) infection is one of the major global health problems resulting in multiple system disorders. The serum uric acid to high density lipoprotein cholesterol ratio (UHR) is a novel index of inflammation and metabolism, but its association with the development of Hp infection is still unclear.

**Materials and methods:**

This is a cross-sectional study involving 2,666 participants, using data from the National Health and Nutrition Examination Survey (NHANES) conducted in the United States. The relationship between UHR and Hp infection was evaluated by multivariate logistic regression and sensitivity analysis to enhance the stability of the results.

**Results:**

Among all individuals, 1,165 were Hp positive (43.7%) and 1,501 were Hp negative (56.3%). After adjustment, there was a positive correlation between UHR and Hp infection (OR = 1.15; 95% CI 1.02–1.30; *P* = 0.020). This association is relatively stable in the subgroup analysis (*P* > 0.05).

**Conclusion:**

There is a positive correlation between the UHR and the development of Hp infection in our study. This non-invasive indicator can improve the ability to monitor Hp infection and may find alternative therapeutic intervention targets.

## Introduction

The Gram-negative microaerophilic bacterium *Helicobacter pylori* (*H. pylori*, Hp) colonizes the human gastric mucosa and forms a chronic infection closely associated with atrophic gastritis, peptic ulcer and gastric cancer and is considered to be one of the most prevalent chronic bacterial infections worldwide (1, 2), affecting nearly half of the world’s population and 35.6% of Americans. Although *H. pylori* infection has decreased in recent decades, it continues to be an important burden of global health given its close association to a range of other diseases (3). *Helicobacter pylori* is listed as the category I carcinogen by the World Health Organization, and is a major factor in the pathogenesis of chronic gastritis, peptic ulcer disease, and noncardiac gastric cancer. Over 75% of duodenal ulcer cases and 17% of gastric ulcer cases are related to infection. In addition, it has been associated with extra-gastrointestinal diseases, including iron deficiency anemia, idiopathic thrombocytopenic purpura (4), cardiovascular disease (5), endocrine dysfunction (6), and Alzheimer’s disease (7). The health burden associated with *H. pylori* infection extends beyond its direct clinical manifestations, affecting patients’ quality of life and imposing considerable healthcare costs due to prolonged treatment and disease management.

Previous studies have shown that *H. pylori* infection is often accompanied by changes in some metabolic indicators and lipid profiles in the blood, for example, a study of black urban Congolese individuals indicate that *H. pylori* infection leads to a significant increase in SUA (8, 9), and another study of adults from Australia and New Zealand indicate that chronic Hp infection reduces the level of HDL in plasma (2, 10). Serum uric acid (SUA) is widely recognized as a biomarker for measuring abnormal renal metabolic function (10). When the concentration of serum uric acid (SUA) increases, it often indicates the occurrence of cell damage (11), and it is closely linked to various disease states such as gout (12), dyslipidemia and cardiovascular disease (13). In contrast, normal high-density lipoprotein cholesterol (HDL) plays an important role in cardiovascular health and overall protection, by promoting reverse transport of cholesterol, as well as having significant anti-inflammatory, antioxidant, and anti-thrombotic capacity (14). Notably, the SUA-to-HDL ratio (UHR) has emerged as a novel biomarker reflecting both inflammatory and metabolic states. Recent studies have demonstrated that UHR is superior to individual SUA or HDL levels in predicting a wide spectrum of diseases, including diabetic nephropathy (15), non-alcoholic fatty liver disease (16), hypertension, thyroid inflammation and other diseases (17). However, its relationship with *H. pylori* infection remains underexplored. This study aims to investigate the association between UHR and *H. pylori* infection, using a large, nationally representative dataset to provide insights into its potential role as a clinical biomarker in this context.

## Materials and methods

### Data sources

**FIGURE 1:**
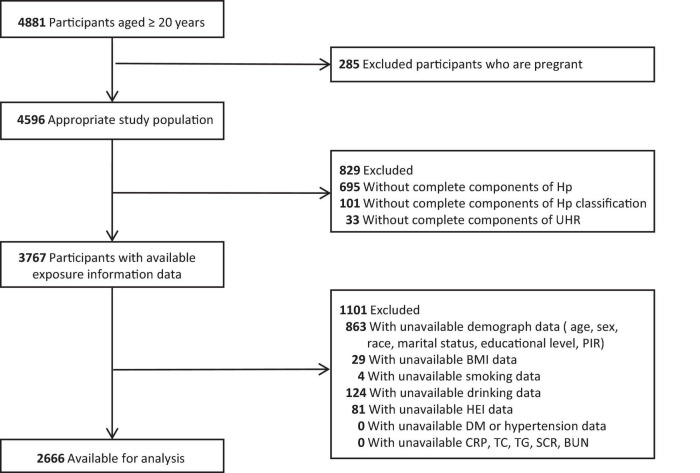
Flow diagram of the sample selection from the National Health and Nutrition Examination Survey (NHANES) 1999-2000. UHR, serum uric acid to high-density lipoprotein cholesterol ratio; Hp, *Helicobacter pylori*; PIR, poverty income ratio; BMI, body mass index; HEI, health eating index; DM, diabetes mellitus; CRP, C-reactive protein; BUN, blood urea nitrogen; SCR, serum creatinine; TC, total cholesterol; TG, triglyceride; SUA, serum uric acid; HDL, high-density lipoprotein.

This study is a representative study based on the National Health and Nutrition Examination Survey (NHANES) database 1999-2000, which is a publicly available database.^1^ NHANES is a nationally representative survey conducted by the Centers for Disease Control and Prevention’s National Center for Health Statistics, designed to assess the health and nutritional status of community adults and children in Central African institutions in the United States through a complex, stratified, multi-stage probability sampling framework (18). All of the study participants signed a written, informed consent form. NHANES Is an open database, so the ethical approval was waived. Of the 4,881 participants included in this survey cycle, individuals were eligible to be included if they had complete data on *H. pylori* serology, lipid profiles, and demographic variables. Participants with missing data or a history of diabetes or hypertension were excluded and the final study cohort was 2666. The selection process of the participants is shown in Figure 1.

### Definitions of Hp seropositivity

In accordance with the NHANES protocol, serum samples were collected via venipuncture, stored at –80°C, and tested for *H. pylori* immunoglobulin G (IgG) antibodies using an enzyme-linked immunosorbent assay (ELISA) kit (Wampole Laboratories, Cranbury, NJ). Seropositivity was defined as an optical density (OD) value ≥ 1.1, while values < 0.9 were classified as seronegative. Ambiguous results (0.9–1.1) were excluded to avoid misleading statistical results.

### Definitions of UHR

Fasting blood samples were analyzed to measure SUA and HDL levels. SUA was quantified using the DxC800 automated chemical analyzer (Beckman Coulter) through uricase-based oxidation, while HDL was measured using enzymatic colorimetric methods. Specific descriptions of the NHANES database can be found on the website (see text footnote 1). The UHR was calculated as the ratio of SUA (mg/dL) to HDL (mg/dL) (19).

### Clinical characteristics and covariates

Covariates in this research included age, sex, race, marital status, education level, poverty-income ratio (PIR), BMI, alcohol consumption, smoking status, alcohol consumption, Healthy Eating Index (HEI), DM, hypertension and relative laboratory parameters, including C-reactive protein (CRP), blood urea nitrogen (BUN), serum creatinine (SCR), total cholesterol (TC), and triglycerides (TG). Among them, ethnicity was classified as Mexican American, non-Hispanic White, non-Hispanic Black, other Hispanic, or other race, while marital status was classified as individuals living with a partner, married, never married, or widowed, divorced or separated. And the education level is classified as lower than high school, high school or equivalent, or college or above. In addition, lifestyle and disease history, including smoking status (current, past, or never), alcohol consumption (current, past or never), HEI calculated by HEI-2015 criterion, DM (yes or no) and hypertension (yes or no) were further collected.

### Statistical analysis

Continuous variables are represented by the median (range of quartiles) or the mean (standard deviation, SD). The UHR was divided into four groups called quintiles. We used the χ 2 test for categorical variables and the Student’s t test or Kruskal–Wallis test for continuous variables to assess the difference between each group. Odds ratios (OR) and 95% confidence intervals (95% CI) of the relationship between UHR and HP infection were determined using logistic regression models. Specifically, the unadjusted analysis model is Model 1. In addition, age, gender and race were briefly adjusted in Model 2. In model 3, we assessed age, gender, race, marital status, educational level, PIR, BMI, smoking status, drinking status, HEI score, DM, hypertension, CRP, BUN, SCR, TC and TG. Furthermore, we performed a subgroup analysis on age group, sex, BMI, DM and hypertension. All analyses were performed using Free Statistics software version 1.9 and the statistical package R.^2^ A two-tail test was performed, and *p* < 0.05 was considered statistically significant.

## Results

### Clinical characteristics of study participants

Among all individual data, 1,165 were positive for Hp infection (43.7%), and 1,501 were negative (56.3%). Table 1 describes the weighted characteristics of the 2,666 subjects based on UHR quartiles. Substantial differences were observed between the UHR quartiles and baseline characteristics. Individuals in the higher quartiles were more likely to be male, smoke more, drink more, and have a higher incidence of hypertension and diabetes mellitus. At the same time, BMI, BUN, SUA, TG, SCR were higher, while HEI and HDL were lower. However, no significant differences were found in age, race, marital status, PIR, TC, or CRP.

**TABLE 1:** Baseline characteristics according to UHR quartiles.

Variables	Total (*n* = 2666)	Quartiles of UHR				*P*
		**1 (*n* = 665)**	**2 (*n* = 668)**	**3 (*n* = 664)**	**4 (*n* = 669)**	
**Age, years, mean ± SD**	50.8 ± 18.3	50.1 ± 17.8	50.1 ± 18.4	51.6 ± 18.4	51.5 ± 18.7	0.252
**Sex, *n* (%)**						<0.001
Female	1326 (49.7)	533 (80.2)	414 (62)	245 (36.9)	134 (20)	
Male	1340 (50.3)	132 (19.8)	254 (38)	419 (63.1)	535 (80)	
**Race, n (%)**						0.009
Mexican American	686 (25.7)	165 (24.8)	176 (26.3)	190 (28.6)	155 (23.2)	
Non-Hispanic Black	454 (17.0)	120 (18)	127 (19)	105 (15.8)	102 (15.2)	
Non-Hispanic White	1271 (47.7)	326 (49)	293 (43.9)	312 (47)	340 (50.8)	
Other Hispanic	173 (6.5)	37 (5.6)	58 (8.7)	37 (5.6)	41 (6.1)	
Other Race	82 (3.1)	17 (2.6)	14 (2.1)	20 (3)	31 (4.6)	
**Marital status, *n* (%)**						<0.001
Living with partner	114 (4.3)	27 (4.1)	29 (4.3)	29 (4.4)	29 (4.3)	
Married	1540 (57.8)	357 (53.7)	348 (52.1)	413 (62.2)	422 (63.1)	
Never married	410 (15.4)	109 (16.4)	97 (14.5)	109 (16.4)	95 (14.2)	
Widowed, divorced, or separated individuals	602 (22.6)	172 (25.9)	194 (29)	113 (17)	123 (18.4)	
**Educational level, *n* (%)**						0.018
Less than high school	977 (36.6)	216 (32.5)	253 (37.9)	251 (37.8)	257 (38.4)	
High school or equivalent	602 (22.6)	138 (20.8)	146 (21.9)	154 (23.2)	164 (24.5)	
Above high school	1087 (40.8)	311 (46.8)	269 (40.3)	259 (39)	248 (37.1)	
**PIR, mean ± SD**	2.6 ± 1.6	2.8 ± 1.7	2.5 ± 1.6	2.5 ± 1.5	2.6 ± 1.6	0.006
**BMI, kg/m2, Mean ± SD**	28.5 ± 6.2	25.9 ± 5.6	28.1 ± 6.0	29.2 ± 5.8	30.7 ± 6.3	<0.001
**Smoking status, *n* (%)**						<0.001
Former	739 (27.7)	137 (20.6)	172 (25.7)	196 (29.5)	234 (35)	
Never	1375 (51.6)	408 (61.4)	352 (52.7)	337 (50.8)	278 (41.6)	
Now	552 (20.7)	120 (18)	144 (21.6)	131 (19.7)	157 (23.5)	
**Drinking status, *n* (%)**						<0.001
Former	557 (20.9)	95 (14.3)	124 (18.6)	159 (23.9)	179 (26.8)	
Never	376 (14.1)	115 (17.3)	109 (16.3)	82 (12.3)	70 (10.5)	
Now	1733 (65.0)	455 (68.4)	435 (65.1)	423 (63.7)	420 (62.8)	
**HEI, Mean ± SD**	50.7 ± 13.1	52.2 ± 13.7	51.6 ± 12.6	50.4 ± 13.1	48.5 ± 12.8	<0.001
**DM, *n* (%)**						0.003
No	2316 (86.9)	602 (90.5)	585 (87.6)	564 (84.9)	565 (84.5)	
Yes	350 (13.1)	63 (9.5)	83 (12.4)	100 (15.1)	104 (15.5)	
**Hypertension, *n* (%)**						<0.001
No	1526 (57.2)	422 (63.5)	397 (59.4)	362 (54.5)	345 (51.6)	
Yes	1140 (42.8)	243 (36.5)	271 (40.6)	302 (45.5)	324 (48.4)	
**CRP, mg/dL, Median (IQR)**	0.2 (0.1, 0.6)	0.2 (0.1, 0.4)	0.2 (0.1, 0.6)	0.2 (0.1, 0.6)	0.3 (0.1, 0.6)	<0.001
**TC, mg/dL, Mean ± SD**	197.7 ± 38.8	199.4 ± 37.8	196.7 ± 40.5	198.4 ± 38.5	196.2 ± 38.4	0.406
**TG, mg/dL, median (IQR)**	118.0 (81.0, 172.0)	86.0 (63.0, 120.0)	104.0 (77.0, 146.2)	132.0 (93.8, 184.2)	161.0 (116.0, 234.0)	<0.001
**BUN, mg/dL, Mean ± SD**	14.9 ± 5.8	13.4 ± 4.4	14.4 ± 5.6	15.1 ± 5.2	16.4 ± 7.1	<0.001
**SCR, mg/dL, Median (IQR)**	0.7 (0.6, 0.9)	0.6 (0.5, 0.7)	0.7 (0.6, 0.8)	0.8 (0.6, 0.9)	0.8 (0.7, 1.0)	<0.001
**HDL, mg/dL, Mean ± SD**	50.2 ± 15.2	66.5 ± 15.2	52.6 ± 9.9	44.9 ± 7.6	36.8 ± 7.4	<0.001
**SUA, mg/dL, Mean ± SD**	5.4 ± 1.5	4.0 ± 0.9	4.9 ± 0.9	5.7 ± 1.0	7.0 ± 1.3	<0.001
**HP infection, *n* (%)**						<0.001
No	1501 (56.3)	411 (61.8)	392 (58.7)	331 (49.8)	367 (54.9)	
Yes	1165 (43.7)	254 (38.2)	276 (41.3)	333 (50.2)	302 (45.1)	

UHR, serum uric acid to high-density lipoprotein cholesterol ratio; Q, quartile; PIR, poverty income ratio; BMI, body mass index; HEI, health eating index; DM, diabetes mellitus; CRP, C-reactive protein; BUN, blood urea nitrogen; SCR, serum creatinine; TC, total cholesterol; TG, triglyceride; SUA, serum uric acid; HDL, high-density lipoprotein; HP, *Helicobacter pylori*.

### Association between UHR and the development of Hp infection

Table 2 provides a multivariate regression analysis between the effects of Hp infection and UHR. In the unadjusted model, UHR was positively correlated with Hp infection (OR = 1.11; 95%CI: 1.03 ∼ 1.20; *P* = 0.006), after adjusting for variables, Model 2 (OR = 1.16; 95%CI: 1.05 ∼ 1.28; *P* = 0.002) and Model 3 (OR = 1.15; 95%CI: 1.02 ∼ 1.30; *P* = 0.020), the correlation between UHR and Hp infection was still positive. In addition, Hp infection rates increased by 3% (*P* = 0.819), 71% (*P* < 0.001), and 45% (*P* = 0.024) in the 2, 3, and 4 quantiles, respectively, compared with the lowest levels of UHR (Q1) in Model 3, and the trend test for Model 3 was *P* = 0.002.

**TABLE 2:** Association between UHR and the development of HP infection.

	Model 1		Model 2		Model 3	
	**OR (95%CI)**	***P*-value**	**OR (95%CI)**	***P*-value**	**OR (95%CI)**	***P*-value**
UHR (per SD increase)	1.11 (1.03∼1.20)	0.006	1.16 (1.05∼1.28)	0.002	1.15 (1.02∼1.30)	0.020
**UHR quartiles**						
Q1	1[Ref]		1[Ref]		1[Ref]	
Q2	1.14 (0.91∼1.42)	0.244	1.06 (0.83∼1.35)	0.656	1.03 (0.79∼1.34)	0.819
Q3	1.63 (1.31∼2.02)	<0.001	1.69 (1.31∼2.18)	<0.001	1.71 (1.29∼2.27)	<0.001
Q4	1.33 (1.07∼1.66)	0.01	1.46 (1.12∼1.91)	0.006	1.45 (1.05∼2.01)	0.024
Trend test		0.001		<0.001		0.002

Model 1: Crude Model. Model 2: Adjusted for age, gender and race. Model 3: Adjusted for age, gender, race, marital status, educational level, PIR, BMI, smoking status, drinking status, HEI score, DM, hypertension, CRP, BUN, SCR, TC, TG. UHR, serum uric acid to high-density lipoprotein cholesterol ratio; HP, *Helicobacter pylori*; PIR, poverty income ratio; BMI, body mass index; HEI, health eating index; DM, diabetes mellitus; CRP, C-reactive protein; BUN, blood urea nitrogen; SCR, serum creatinine; TC, total cholesterol; TG, triglyceride.

**FIGURE 2:**
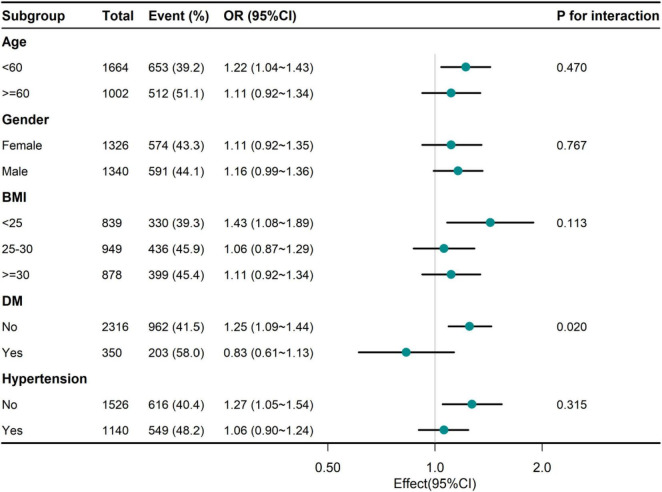
Association between NHHR and Hp infection of adults in different subgroups. UHR, serum uric acid to high-density lipoprotein cholesterol ratio; Hp, *Helicobacter pylori*; BMI, body mass index; DM, diabetes mellitus.

Hierarchical analysis of additional variables. As shown in Figure 2, we performed a stratified analysis across several subgroups to assess the potential impact of the relationship between Hp infection and UHR. When stratified by age, sex, BMI, and hypertension, no significant interactions were found in any subgroup. Considering multiple tests, a *P*-value of DM interaction less than 0.05 May not be statistically significant.

## Discussion

This study is among the first to explore the association between the SUA-to-HDL ratio (UHR) and *H. pylori* infection, revealing a significant positive correlation that persists after adjusting for multiple demographic, metabolic, and inflammatory confounders. These findings highlight the potential of UHR as a novel biomarker reflecting the interplay between metabolic dysregulation and chronic infection. While prior research has independently linked elevated SUA and reduced HDL to inflammation and metabolic dysfunction, our results underscore the clinical utility of UHR as a composite index that integrates these factors.

The data of this study showed that the high quartile UHR individuals were more male, smokers, drinkers and hypertensive diabetic patients, and had lower HEI levels and higher TG, BUN and SCR levels, which were consistent with the results of previous studies to varying degrees (16, 20). In the association between UHR and Hp infection, we found that UHR was positively correlated with Hp infection regardless of model adjustment. After adjusting for multiple confounders, the risk of Hp infection increased by 15% for each SD increase in UHR. (21) Furthermore, subgroup analyses indicate that this relationship is robust across diverse demographic and clinical subpopulations, suggesting its generalizability and relevance to clinical practice. The marginal interaction observed in the diabetes subgroup, while intriguing, requires further investigation to clarify its biological significance.

The observed association between UHR and *H. pylori* infection may reflect underlying pathophysiological mechanisms shared by metabolic and inflammatory processes (22). Elevated SUA, a byproduct of purine metabolism, is known to promote oxidative stress and low-grade systemic inflammation, both of which can facilitate (23–25). *H. pylori* colonization and persistence in the gastric mucosa (26). Moreover, reduced HDL levels impair cholesterol transport and endothelial protection, which may compromise the host’s immune defense against bacterial infection (27–29). Together, these metabolic alterations may create a permissive environment for *H. pylori* to establish chronic infection, contributing to the observed association. Besides, for the interaction observed in the diabetes subgroup, we hypothesized that this may be due to increased levels of inflammation and metabolic disorders in diabetic patients themselves, which in turn disrupt the normal metabolic pathways of SUA and HDL in humans (30), thus reducing the negative effect of UHR. (23, 31)

The implications of these findings extend beyond *H. pylori* infection, given the established role of UHR in predicting cardiovascular and metabolic diseases. The overlap between metabolic and infectious pathways emphasizes the importance of a multidisciplinary approach to disease prevention and management. For instance, targeting UHR through dietary and pharmacological interventions may not only improve metabolic health but also mitigate the risk of chronic infections, including *H. pylori*. This hypothesis warrants exploration in future interventional studies.

The study has several limitations. First, this cross-sectional study was unable to establish a causal relationship between serum UHR levels and Hp infection, and the absence of follow-up data limits understanding of how UHR changes with Hp treatment or progression. Besides, our study does not explore the biological mechanisms underlying the association between UHR and Hp infection, which needs further investigation. Moreover, despite adjustments, residual confounding factors, such as dietary habits, physical activity, or genetic predisposition, may influence the results. Finally, because the NHANES database represents only the U.S. population, the results may have some limitations worldwide. To further confirm our conclusions, a prospective cohort study with a larger sample size is needed.

In conclusion, our study identifies UHR as a promising biomarker for *H. pylori* infection, reflecting its potential role at the intersection of metabolic dysfunction and chronic inflammation. Future research should focus on elucidating the mechanistic underpinnings of this relationship, exploring its clinical utility in risk stratification and targeted interventions, and validating these findings in diverse populations. By integrating metabolic and infectious pathways, UHR offers a novel perspective on the complex interplay between chronic diseases, advancing our understanding of their shared pathophysiology.

## Data Availability

The original contributions presented in this study are included in this article/supplementary material, further inquiries can be directed to the corresponding author.

## Footnotes

1
https://www.cdc.gov/nchs/nhanes/

2 http://www.R-project.org, R Foundation

## References

[1] Sidebotham R Baron J. Hypothesis: *Helicobacter pylori*, urease, mucus, and gastric ulcer. *Lancet.* (1990) 335(8683):193–5. 10.1016/0140-6736(90)90279-e 1967668

[2] Malfertheiner P Camargo M El-Omar E Liou J Peek R Schulz C *Helicobacter pylori* infection. *Nat Rev Dis Primers.* (2023) 9(1):19. 37081005 10.1038/s41572-023-00431-8 PMC11558793

[3] Gatta L Vakil N Vaira D Scarpignato C. Global eradication rates for *Helicobacter pylori* infection: Systematic review and meta-analysis of sequential therapy. *Bmj.* (2013) 347:f4587. 10.1136/bmj.f4587 23926315 PMC3736972

[4] Robinson K Atherton J. The spectrum of *Helicobacter*-mediated diseases. *Annu Rev Pathol.* (2021) 16:123–44. 33197219 10.1146/annurev-pathol-032520-024949

[5] Li W Zhang J Ma J Li Z Zhang L Zhang Y Effects of *Helicobacter pylori* treatment and vitamin and garlic supplementation on gastric cancer incidence and mortality: Follow-up of a randomized intervention trial. *Bmj.* (2019) 366:l5016. 10.1136/bmj.l5016 31511230 PMC6737461

[6] McCracken K Catá E Crawford C Sinagoga K Schumacher M Rockich B Modelling human development and disease in pluripotent stem-cell-derived gastric organoids. *Nature.* (2014) 516(7531):400–4. 25363776 10.1038/nature13863 PMC4270898

[7] Xie J Cools L Van Imbschoot G Van Wonterghem E Pauwels M Vlaeminck I *Helicobacter pylori*-derived outer membrane vesicles contribute to Alzheimer’s disease pathogenesis via C3-C3aR signalling. *J Extracell Vesicles.* (2023) 12(2):e12306. 10.1002/jev2.12306 36792546 PMC9931688

[8] Feig D Kang D Johnson R. Uric acid and cardiovascular risk. *N Engl J Med.* (2008) 359(17):1811–21. 18946066 10.1056/NEJMra0800885 PMC2684330

[9] Longo-Mbenza B Nsenga J Mokondjimobe E Gombet T Assori I Ibara J *Helicobacter pylori* infection is identified as a cardiovascular risk factor in Central Africans. *Vasc Health Risk Manag.* (2012) 6:455–61. 10.2147/VHRM.S28680 22923995 PMC3423148

[10] Badve S Pascoe E Tiku A Boudville N Brown F Cass A Effects of allopurinol on the progression of chronic kidney disease. *N Engl J Med.* (2020) 382(26):2504–13. 32579811 10.1056/NEJMoa1915833

[11] Iracheta-Vellve A Petrasek J Satishchandran A Gyongyosi B Saha B Kodys K Inhibition of sterile danger signals, uric acid and ATP, prevents inflammasome activation and protects from alcoholic steatohepatitis in mice. *J Hepatol.* (2015) 63(5):1147–55. 10.1016/j.jhep.2015.06.013 26100496 PMC4615393

[12] Dalbeth N Gosling A Gaffo A Abhishek A. Gout. *Lancet.* (2021) 397(10287):1843–55. 33798500 10.1016/S0140-6736(21)00569-9

[13] Mackenzie I Ford I Nuki G Hallas J Hawkey C Webster J Long-term cardiovascular safety of febuxostat compared with allopurinol in patients with gout (FAST): A multicentre, prospective, randomised, open-label, non-inferiority trial. *Lancet.* (2020) 396(10264):1745–57. 10.1016/S0140-6736(20)32234-0 33181081

[14] Magnussen C Ojeda F Leong D Alegre-Diaz J Amouyel P Aviles-Santa L Global effect of modifiable risk factors on cardiovascular disease and mortality. *N Engl J Med.* (2023) 389(14):1273–85. 37632466 10.1056/NEJMoa2206916 PMC10589462

[15] Aktas G Yilmaz S Kantarci D Duman T Bilgin S Balci S Is serum uric acid-to-HDL cholesterol ratio elevation associated with diabetic kidney injury? *Postgrad Med.* (2023) 135(5):519–23. 10.1080/00325481.2023.2214058 37170820

[16] Xie Y Huang K Zhang X Wu Z Wu Y Chu J Association of serum uric acid-to-high-density lipoprotein cholesterol ratio with non-alcoholic fatty liver disease in American adults: A population-based analysis. *Front Med (Lausanne).* (2023) 10:1164096. 10.3389/fmed.2023.1164096 37256087 PMC10225665

[17] Li Z Liu Q Yao Z. The serum uric acid-to-high-density lipoprotein cholesterol ratio is a predictor for all-cause and cardiovascular disease mortality: A cross-sectional study. *Front Endocrinol (Lausanne).* (2024) 15:1417485. 10.3389/fendo.2024.1417485 PMC11427315 39345882

[18] Curtin L Mohadjer L Dohrmann S Kruszon-Moran D Mirel L Carroll M National health and nutrition examination survey: Sample design, 2007-2010. *Vital Health Stat.* (2013) 160:1–23. 25090039

[19] Chen Z Cheang I Qu Q Zhu X Fu Y Gao R Associations of serum uric acid-to-high density lipoprotein cholesterol ratio with age-related diseases and mortality among older population in the United States. *Arch Gerontol Geriatr.* (2024) 130:105707. 10.1016/j.archger.2024.105707 39626425

[20] Huang X Hu L Tao S Xue T Hou C Li J. Relationship between uric acid to high-density cholesterol ratio (UHR) and circulating α-klotho: Evidence from NHANES 2007-2016. *Lipids Health Dis.* (2024) 23(1):244. 10.1186/s12944-024-02234-6 39123222 PMC11312937

[21] Danesh J Peto R. Risk factors for coronary heart disease and infection with *Helicobacter pylori*: Meta-analysis of 18 studies. *Bmj.* (1998) 316(7138):1130–2. 10.1136/bmj.316.7138.1130 9552950 PMC28515

[22] Kocak M Aktas G Erkus E Sincer I Atak B Duman T. Serum uric acid to HDL-cholesterol ratio is a strong predictor of metabolic syndrome in type 2 diabetes mellitus. *Rev Assoc Med Bras (1992).* (2019) 65(1):9–15. 10.1590/1806-9282.65.1.9 30758414

[23] Du L Zong Y Li H Wang Q Xie L Yang B Hyperuricemia and its related diseases: Mechanisms and advances in therapy. *Signal Transduct Target Ther.* (2024) 9(1):212. 10.1038/s41392-024-01916-y 39191722 PMC11350024

[24] Kisker C Schindelin H Baas D Rétey J Meckenstock R Kroneck PMA. structural comparison of molybdenum cofactor-containing enzymes. *FEMS Microbiol Rev.* (1998) 22(5):503–21. 9990727 10.1111/j.1574-6976.1998.tb00384.x

[25] Crawley W Jungels C Stenmark K Fini MAU-. shaped association of uric acid to overall-cause mortality and its impact on clinical management of hyperuricemia. *Redox Biol.* (2022) 51:102271. 10.1016/j.redox.2022.102271 35228125 PMC8889273

[26] Kusters J van Vliet A Kuipers E. Pathogenesis of *Helicobacter* pylori infection. *Clin Microbiol Rev.* (2006) 19(3):449–90. 16847081 10.1128/CMR.00054-05 PMC1539101

[27] Riwanto M Rohrer L von Eckardstein A Landmesser U. Dysfunctional HDL: From structure-function-relationships to biomarkers. *Handb Exp Pharmacol.* (2015) 224:337–66. 25522994 10.1007/978-3-319-09665-0_10

[28] Vitali C Wellington C Calabresi L. HDL and cholesterol handling in the brain. *Cardiovasc Res.* (2014) 103(3):405–13. 24907980 10.1093/cvr/cvu148

[29] Poliakova T Wellington C. Roles of peripheral lipoproteins and cholesteryl ester transfer protein in the vascular contributions to cognitive impairment and dementia. *Mol Neurodegener.* (2023) 18(1):86. 10.1186/s13024-023-00671-y 37974180 PMC10652636

[30] Ishibashi T Kaneko H Matsuoka S Suzuki Y Ueno K Ohno R HDL cholesterol and clinical outcomes in diabetes mellitus. *Eur J Prev Cardiol.* (2023) 30(8):646–53. 36738171 10.1093/eurjpc/zwad029

[31] Marsche G Saemann M Heinemann A Holzer M. Inflammation alters HDL composition and function: Implications for HDL-raising therapies. *Pharmacol Ther.* (2013) 137(3):341–51. 10.1016/j.pharmthera.2012.12.001 23246719

